# AmpliCoV: Rapid Whole-Genome Sequencing Using Multiplex PCR Amplification and Real-Time Oxford Nanopore MinION Sequencing Enables Rapid Variant Identification of SARS-CoV-2

**DOI:** 10.3389/fmicb.2021.651151

**Published:** 2021-07-01

**Authors:** Annika Brinkmann, Sophie-Luisa Ulm, Steven Uddin, Sophie Förster, Dominique Seifert, Rainer Oehme, Merle Corty, Lars Schaade, Janine Michel, Andreas Nitsche

**Affiliations:** ^1^Highly Pathogenic Viruses, Centre for Biological Threats and Special Pathogens, WHO Reference Laboratory for SARS-CoV-2 and WHO Collaborating Centre for Emerging Infections and Biological Threats, Robert Koch Institute, Berlin, Germany; ^2^Landesgesundheitsamt Baden-Württemberg, Stuttgart, Germany; ^3^LADR Laboratory Group Dr. Kramer & Colleagues, Geesthacht, Germany

**Keywords:** SARS-CoV-2, MinION, whole-genome sequencing, ampliseq, mutation detection

## Abstract

Since the emergence of the Severe Acute Respiratory Syndrome Coronavirus-2 (SARS-CoV-2) in December 2019, the scientific community has been sharing data on epidemiology, diagnostic methods, and whole-genomic sequences almost in real time. The latter have already facilitated phylogenetic analyses, transmission chain tracking, protein modeling, the identification of possible therapeutic targets, timely risk assessment, and identification of novel variants. We have established and evaluated an amplification-based approach for whole-genome sequencing of SARS-CoV-2. It can be used on the miniature-sized and field-deployable sequencing device Oxford Nanopore MinION, with sequencing library preparation time of 10 min. We show that the generation of 50,000 total reads per sample is sufficient for a near complete coverage (>90%) of the SARS-CoV-2 genome directly from patient samples even if virus concentration is low (Ct 35, corresponding to approximately 5 genome copies per reaction). For patient samples with high viral load (Ct 18–24), generation of 50,000 reads in 1–2 h was shown to be sufficient for a genome coverage of >90%. Comparison to Illumina data reveals an accuracy that suffices to identify virus mutants. AmpliCoV can be applied whenever sequence information on SARS-CoV-2 is required rapidly, for instance for the identification of circulating virus mutants.

## Introduction

In November 2002, an outbreak of atypical pneumonia was reported in Guangdong, Southern China (Zhao et al., 2003; Zhong et al., 2003). On 16 April 2003, the continuing outbreak was announced by the World Health Organization to be caused by a novel coronavirus (CoV), termed Severe Acute Respiratory Syndrome Coronavirus 1 (SARS-CoV; World Health Organization [WHO], 2003b). The first whole-genome sequences of the novel virus were made available on 30 May 2003, more than 6 months after the first reported cases (Marra et al., 2003; Rota et al., 2003). Until then, the viral disease had caused more than 8,000 infections and over 750 deaths (World Health Organization [WHO], 2003a) and not much was known about the virus’ phylogeny, origin, and routes of transmission.

Seventeen years after the emergence of the first human-pathogenic CoV of the 21st century, the world is being challenged by a novel CoV similar to SARS-CoV, termed SARS-CoV-2. Cases of patients with unknown pneumonia in Wuhan, China, were officially reported on 31 December 2019 (The Government of the Hong Kong Special Administrative Region, 2019). Only 1 week later, on 8 January 2020, the novel CoV was identified from patient samples, followed by the release of the first whole-genome sequence of the virus on the following day (Holmes, 2020; Khan, 2020; World Health Organization [WHO], 2020). As of 17 March 2021, 119,960,700 diagnosed cases worldwide with 2,656,822 deaths from SARS-CoV-2 infections have been reported^1^.

The rapid discovery of SARS-CoV-2 and the prompt publication of whole-genome sequences demonstrate that thanks to modern technologies the response to disease outbreaks has improved markedly since the SARS-CoV outbreak. To date, more than 792,347 SARS-CoV-2 genome sequences have been published on GISAID (Global Initiative on Sharing all Influenza Data; GISAID, 2020). The genomic data have enabled extensive analyses of phylogenetics and transmission chains, including the report of a proposed single introduction into the human population, probably having occurred in Wuhan in November or early December 2019 (Andersen et al., 2020).

The rapid publication and analysis of full genome sequences of SARS-CoV-2 have been achieved with the development of High-Throughput Sequencing (HTS). The most common platforms for HTS are the Illumina sequencing devices. Generated sequences from clinical samples are accurate and of high quality, but costs for acquisition of the device and reagents as well as for data storage are high. In addition, sequence information for the host is usually generated by shotgun approaches, conflicting with personal data protection. The Oxford Nanopore MinION (Oxford Nanopore Technologies, Oxford, United Kingdom) is a portable long-read sequencing device that offers sequencing in real time. The low acquisition and reagent costs have enabled sequencing for numerous smaller laboratories and even under field conditions. However, compared with Illumina sequencing, the quality of the sequences is low and can challenge the identification of Single Nucleotide Polymorphisms, insertions, and deletions in sequenced genomes. Such difficulties can be overcome by a high read coverage of the genome, which can be achieved by amplicon sequencing as has been performed during the Ebola virus epidemic in 2014, for Zika virus, and more recently for SARS-CoV-2^2^ (Quick et al., 2016, 2017; Freed et al., 2020; Gohl et al., 2020; Hourdel et al., 2020). Moreover, specific amplification of the viral genome excludes data protection problems because the host genome is not elucidated simultaneously.

In this study, we evaluate a two-reaction multiplex PCR with 113 and 111 primers, which can be used for amplification and whole-genome sequencing of SARS-CoV-2 in less than 3 h from sample to result on the Oxford Nanopore MinION. The method can also be performed with a rapid library preparation time of only 10 min. After establishment of AmpliCoV using SARS-CoV-2 propagated in cell culture and samples obtained through the INSTAND external quality assurance exercise for molecular diagnostics of SARS-CoV-2, we analyzed various clinical specimens to prove the applicability of AmpliCoV in routine diagnostics, to elucidate transmission chains, and to identify virus mutants that may contribute to more efficient transmissibility.

## Materials and Methods

### Primer Design and Evaluation

Primers have been designed by using Primer3 v2.3.7 (Untergasser et al., 2012). For the primer design, all references for SARS-CoV-2 available on GISAID (23 January 2020) and the NCBI RefSeq reference for SARS-CoV-1 (NC_004718) were aligned by using MAFFT v7.450. If possible, primers were designed on regions conserved between all SARS-CoV-2 and SARS-CoV sequences to enable amplification also at regions with possibly arising mutations. We designed the primers to cover the whole genome with tiled primers separated into two pools (113 and 111 primers), with amplicon sizes between 199 and 775 bp (average 415 bp), with overlaps between the amplicons between 7 and 258 bp (Supplementary Material and Table 2), excluding the 3′ and 5′ ends of the genome (bases 0–68 and 29,830–29,904). All primers have been selected based on their melting temperature Tm (minimum Tm 59°C, maximum Tm 61°C, and optimal Tm 60°C) and resulting amplicon length (250–650 bp). Primer sequences are shown in Supplementary Table 1.

### Evaluation of AmpliCoV Sequencing With Cell Culture-Propagated Virus Strains and Human Clinical Specimens

The performance of the AmpliCoV sequencing of SARS-CoV-2 was evaluated with selected virus strains. Nucleic acids from cultured SARS-CoV-2 strain hCoV-19/Italy/INMI1-isl/2020 (National Institute for Infectious Diseases, Rome, Italy, GISAID Accession EPI_ISL_410545), SARS-CoV-2 strain hCoV-19/Germany/BY-ChVir-929/2020 (Institut für Mikrobiologie der Bundeswehr, Munich, Germany, GISAID Accession EPI_ISL_406862), and strain hCoV-19/Japan/TY-WK-521/2020 (Laboratory of Neurovirology, National Institute of Infectious Diseases, Toyama, Tokyo, Japan, GISAID Accession EPI_ISL_408667) were extracted with the QIAamp Viral RNA Mini Kit (Qiagen, Hilden, Germany). cDNA synthesis was performed according to the SuperScript IV Reverse Transcriptase protocol (Thermo Fisher Scientific, Waltham, MA, United States) with random hexamers (65°C for 5 min and 23°C for 10 min), followed by incubation at 55°C for 10 min and inactivation at 80°C for 10 min. Relative genome concentration of all strains was determined by specific quantitative real-time PCR (Michel et al., 2021). The performance of the AmpliCoV sequencing was further tested on clinical specimens (*n* = 36) from patients with a clinical and laboratory diagnosis of COVID-19. All specimens are listed in Tables 1–3. Three additional specimens were analyzed to prove transmission chains and to identify virus mutants, respectively. Ethics approval was obtained from the Berliner Ärztekammer (#Eth 20/40).

**TABLE 1:** AmpliCoV sequencing of SARS-CoV-2-positive clinical specimens from Germany, direct comparison of rapid library and ligation library.

Sample ID	Ct	Percentage genome coverage and time of sequencing with depth coverage > 10 after generation of 50,000 reads per sample			
		Percentage genome coverage (length)	Time of sequencing (hours)	Percentage genome coverage (length)	Time of sequencing (hours)
				
		Rapid library*		Ligation library*	
AC_01	18.1	98.4	02:42:41	97.3	00:53:15
AC_02	18.4	97.3	01:45:58	96.4	00:40:15
AC_03	24.7	94.4	02:42:35	91.9	00:31:16
AC_04	23.3	96.0	02:41:20	93.6	00:44:32
AC_05	17.7	96.9	01:44:46	95.9	00:40:09
AC_06	21.9	96.0	01:57:23	94.1	00:42:05
AC_07	23.2	95.0	02:33:40	95.0	00:35:09
AC_08	22.1	92.0	01:39:19	92.6	00:40:29

*Multiplexing of 11 samples on one flow cell.

**TABLE 2:** AmpliCoV Sequencing of SARS-CoV-2-positive clinical specimens from Germany, rapid library.

Sample ID	Ct		Percentage genome coverage with depth coverage > 10 after generation of 50,000 reads per sample	
		Percentage genome coverage (length)	Time of sequencing (hours)	No of samples on flow cell
ACR_01	18.6	98.4	02:24:53	6
ACR_02	19.1	98.6	01:51:49	6
ACR_03	20.1	99.2	02:27:03	11
ACR_04	20.5	98.1	01:39:19	11
ACR_05	20.5	99.2	02:20:57	11
ACR_06	21.4	98.8	02:01:01	6
ACR_07	23.1	98.3	02:47:14	11
ACR_08	26.2	96.7	01:55:32	6
ACR_09	30.0	93.4	02:36:49	11
ACR_10	31.0	96.9	13:56:26	11

**TABLE 3:** AmpliCoV Sequencing of SARS-CoV-2-positive clinical specimens from Germany, ligation library.

Sample ID	Ct		Percentage genome coverage with depth coverage > 10 after generation of 50,000 reads per sample	
		Percentage genome coverage (length)	Time of sequencing (hours)	No of samples on flow cell
ACL_01	18.5	99.1	01:13:18	11
ACL_02	19.6	99.2	01:21:10	11
ACL_03	19.9	99.3	01:34:57	11
ACL_04	20.5	99.2	01:07:23	11
ACL_05	22.1	99.1	01:11:25	11
ACl_06	22.0	99.1	01:08:16	11
ACl_07	25.3	99.2	01:35:11	15
ACl_08	26.1	99.1	01:44:12	15
ACl_09	26.9	99.0	01:08:26	15
ACl_10	28.1	98.6	00:09:34	5
ACl_11	29.6	97.4	01:58:46	20
ACl_12	29.7	97.3	03:29:22	11
ACl_13	30.1	95.3	05:55:00	20
ACl_14	31.0	97.4	00:56:08	8
ACl_15	31.0	96.6	00:42:21	8
ACl_16	32.0	96.6	01:27:33	8
ACl_17	34.5	89.9	11:50:54	11
ACl_18	35.0	92.2	16:08:41	8

### Evaluation of AmpliCoV Sequencing via Samples of an INSTAND Quality Assurance Exercise

The AmpliCoV method was further tested with samples for a national quality assurance exercise provided by INSTAND e.V. INSTAND e.V. offers exercises for quality assurance for medical laboratories across Germany. This exercise focused on the genomic detection of SARS-CoV-2 and contained inactivated samples for sensitivity (SARS-CoV-2 in different concentrations) and specificity assessment (e.g., other coronaviruses).

### PCR Amplification

The samples were amplified in two reactions with primer pool A and primer pool B designed for whole-genome amplification of SARS-CoV-2 with the following PCR conditions for each primer pool: 3 μl of viral cDNA,1.6 μl of primer pool (100 μM), 0.2 mM dNTP (Invitrogen, Karlsruhe, Germany), 4 μl of 10 × Platinum Taq buffer, 2 mM MgCl_2_, and 5 U Platinum Taq polymerase (Invitrogen) with added water to a final volume of 25 μl. Cycling conditions were 94°C for 5 min, 45 amplification cycles at 94°C for 20 s, 65°C for 30 s, 72°C for 30 s, and a final extension step for 5 min (at 72°C). Thermal cycling was performed in an Eppendorf Mastercycler Pro (Eppendorf Vertrieb Deutschland, Wesseling-Berzdorf, Germany) with a total runtime of 84 min.

### Library Preparation and NGS Sequencing

For MinION sequencing, amplicons were processed for nanopore sequencing via MinION (Oxford Nanopore Technologies). The libraries were prepared by using the Rapid Barcoding Kit, SQK-RBK004 (Oxford Nanopore Technologies), and the Ligation Sequencing Kit, LSK-109. Before library preparation, the PCR reactions were purified with AMPure XP Beads in a ratio of 1:1 (Beckman Coulter Diagnostics, Brea, CA, United States). The barcoded samples were then combined and concentrated with AMPure XP Beads in a ratio of 1:1. Subsequently, the libraries were loaded onto Oxford Nanopore MinION SpotON Flow Cells FLO-MIN106D, R9.4.1 (Oxford Nanopore Technologies). The cell culture-propagated virus strains were prepared with the Rapid Barcoding Kit and sequenced on single SpotON Cells each; the patient samples were prepared with the Rapid Barcoding Kit and Ligation Sequencing Kit, barcoded, combined, and sequenced on one SpotON Cell; the samples of the INSTAND quality assessment study were prepared with the Ligation Sequencing Kit, barcoded, and sequenced on a SpotON Flow Cell. Since patient samples were sequenced as part of the routine virus diagnostics, different levels of multiplexing barcodes were applied based on the number of samples, and Flow Cells with variable active pore numbers were used.

For Illumina sequencing, cDNA and double strand cDNA synthesis was performed using Superscript IV reverse transcriptase (Thermo Fisher Scientific, Darmstadt, Germany) and Non-directional RNA Second Strand Synthesis Module (New England Biolabs, Ipswich, MA, United States). Sequencing was performed on the Illumina MiSeq with the Paragon CleanPlex assay (Paragon Genomics, Hayward, CA, United States).

### Bioinformatics Analysis

The Fast5 files generated during sequencing were basecalled to FastQ files by using Guppy v.3.4.5 (Oxford Nanopore Technologies) on the MinION IT device (MNT-001). Furthermore, barcoded reads were demultiplexed and identified barcodes were clipped. The reads separated by barcode were then further trimmed with Porechop^3^, removing residual barcode and adapter sequences and separating chimeric reads into separate reads. The trimmed FastQ reads for each sample were aligned to the used strain reference if known; for patient samples strain Wuhan-Hu-1 (Accession NC_045512) was used as a reference with Guppy v.3.4.5. Based on the resulting BAM files, primer sequences were soft clipped with bamclipper v.1.1.1 and all soft clippings from the BAM file were removed with custom python scripts. The assembled file was then polished and the consensus sequence was called using medaka v. 1.2.3^4^. Furthermore, the resulting consensus sequences were masked at regions with coverage depth <10, based on bedtools v2.26.0 genomecov histograms^5^ with custom python scripts. Ambiguities and indels in homopolymer regions were corrected manually, based on the reference genome. For each sample, 50,000 reads were analyzed.

All sequence data is deposited under the project number PRJEB42647 at the European Nucleotide Archive.

## Results

### Whole-Genome Sequencing of Cell Culture-Propagated SARS-CoV-2

**FIGURE 1:**
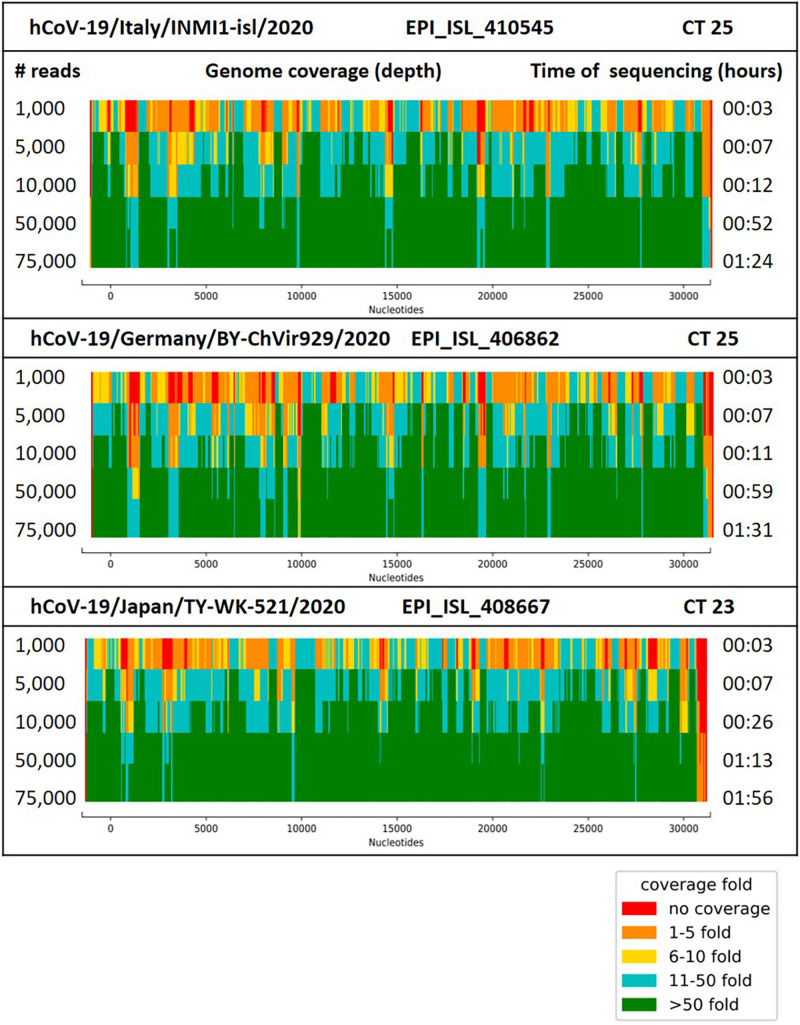
Coverage (depth) for the genome of SARS-CoV-2 strain hCoV-19/Italy/INMI1-isl/2020, hCoV-19/Germany/BY-ChVir929/2020 and hCoV-19/Japan/TY-WK-521/2020 after generation of 10,000–100,000 reads with the AmpliCoV approach. Ct values reflect the amount of 3 μl of cDNA, transcribed from 1.5 μl of viral RNA.

Sequencing of the amplified DNA from the isolates hCoV-19/Germany/BY-ChVir-929/2020, hCoV-19/Italy/INMI1-isl/2020, and hCoV-19/Japan/TY-WK-521/2020 was performed on Oxford Nanopore SpotON (478–654 active pores) Flow Cells. Whole-genome sequencing of SARS-CoV-2 could be performed for all three isolates with >98% genome coverage after generation of 75,000 reads, with a coverage depth >10 for all regions on a SpotON Flow Cell (01:24-01:56 h of sequencing time, Figure 1). Although MinION-generated sequences are prone to high error rates, the identified genome sequences were 100% identical to the reference genomes, except for strain hCoV-19/Italy/INMI1-isl/2020, which had one SNP at genome position 2269, which was confirmed after repeated sequencing. The high accuracy could be shown for sample preparation with the Ligation Kit as well as the Rapid Kit where crossover of barcodes can be observed because of less stringent demultiplexing with only a single barcode being present on the read.

To analyze the performance of AmpliCoV sequencing with potentially cross-reacting coronaviruses, SARS-CoV, HKU1, NL63, OC43, and 229E were amplified with the AmpliCoV primers. No amplification was shown for HKU1, NL63, OC43, and 229E. Although for SARS-CoV the amplification failed for some genome regions with no matching primers, those genomic regions with up to two mismatches between primers and genome sequence were still amplified with low coverage, demonstrating that AmpliCoV could even be performed with increasing genome mutation of SARS-CoV-2. However, after sequencing, all amplicons of SARS-CoV could be distinguished from SARS-CoV-2, eliminating wrong identification and false positives.

### Evaluation on INSTAND External Quality Assurance Exercise Samples

The AmpliCoV method was further tested with samples provided by INSTAND e.V. for quality assurance for SARS-CoV-2 diagnostics in medical laboratories across Germany. All seven samples and a negative control were barcoded and prepared with the LSK-109 Ligation Kit for library preparation and sequenced on a SpotON Flow Cell. The results were identical for all samples tested with specific SARS-CoV-2 qPCRs and AmpliCoV. Genome coverage between 94% (Ct 28.2) and 99.6% (Ct 21.4) could be achieved for samples positive for SARS-CoV-2 (Ct 21.4–31.3; Table 4). No reads for OC43 or 229E were identified.

**TABLE 4:** Results of AmpliCoV and specific qPCRs of the INSTAND exercise for quality assurance for SARS-CoV-2 diagnostics.

Sample	Result qPCR	Ct qPCR	AmpliCoV	Genome coverage, SpotON flow cell (depth ≥ 10)
INSTAND-340059	SARS-CoV-2	21.4	SARS-CoV-2	99.6%
INSTAND-340060	OC43	25.3	Negative	
INSTAND-340061	SARS-CoV-2	31.3	SARS-CoV-2	95.3%
INSTAND-340062	Negative		Negative	
INSTAND-340063	SARS-CoV-2	24.6	SARS-CoV-2	99.1%
INSTAND-340064	SARS-CoV-2	28.2	SARS-CoV-2	94.0%
INSTAND-340065	229E	24.7	Negative	
NK	Negative		Negative	

### Whole-Genome Sequencing of SARS-CoV-2 From Human Clinical Specimens of Varying Virus Load

For further validation of AmpliCoV sequencing, samples from patients diagnosed with SARS-CoV-2 by PCR were amplified, prepared with the Rapid Barcoding and Ligation Sequencing Kit, and sequenced on Oxford Nanopore SpotON Flow Cells until generation of 50,000 reads per sample (Tables 1–3). The consensus sequences and percentage genome coverages were calculated for regions with depth coverage > 10. For low Ct values (Ct 17–23), sequencing with both Rapid and Ligation Sequencing Kit could generate genomes with genome percentage coverages of 92.6–99.3% (Ligation Sequencing, Tables 1, 3) and 92.0–99.2% (Rapid Sequencing, Tables 1, 3). With a Ct range of 24–29, genome percentage coverages from 91.9–99.2% were achieved (Ligation Sequencing, Tables 1, 3). With the Rapid Sequencing Kit, no samples with Ct > 31 could be sequenced, whereas sequencing of samples with Ct of up to 35.0 was possible with the Sequencing by Ligation Kit (genome percentage coverage of 92.2%, Table 3). However, sequencing times increased from 01:13 h (Ct 18.5) and 01:27 (Ct 32.0) up to 11:50 h (Ct 34.5) and 16:08 h (Ct 35.0). In direct comparison of both methods for library preparation, sequencing times were longer for the Rapid Library Kit, whereas genome percentage coverage was slightly, but not significantly higher (Table 1). The genomes generated by the Rapid and Ligation Sequencing Kit were identical in direct comparison, except for single indels in homopolymer regions, which were corrected manually.

**FIGURE 2:**
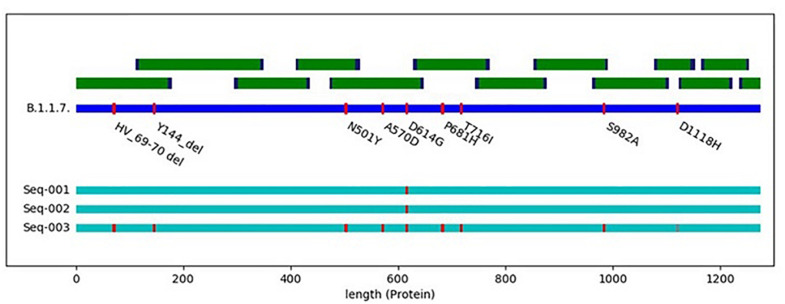
Schematic illustration of the SARS-CoV-3 spike protein with all main mutations of the spike protein (deletions 69–70, deletion 144, N501Y, A570D, D614G, P681H, T716I, S982A, and D1118H) in reference to hCoV-19/England/CAMC-C44C79/2020| EPI_ISL_718552| 2020-12-10. Seq-001 to Seq-003 represent clinical specimens carrying different mutations in the S gene.

Following discussions about SARS-CoV-2 mutants circulating in minks in Denmark and elsewhere that may represent escape mutants with respect to the newly developed vaccines, we could show by *in silico* analysis that AmpliCoV could rapidly and reliably show respective mutations in short time (data not shown). Similarly, the recently described SARS-CoV-2 mutants from the United Kingdom (for example the B.1.1.7. variant), displaying a more efficient transmissibility, can be identified by AmpliCoV. Figure 2 comprises the S gene sequence of SARS-CoV-2 in relation to the primer sequences used with AmpliCov for the S gene region. AmpliCoV sequencing of three patient specimens (Ct values Seq-001 = 21.4, Seq-002 = 20.9, and Seq-003 = 32.3) suspected to be B.1.1.7. variants resulted in almost complete coverage of the S gene sequence (genome percentage coverage with coverage depth > 10 of 100, 96.2, and 85.2%) with identification of all possible sites of mutation except for mutation D1118H for sequence Seq-003. For Seq-003, taken from a patient after returning from London, United Kingdom, AmpliCoV sequencing enabled identification of 8 out of 9 mutations described for B.1.1.7. in the S gene. For sequences Seq-001 and Seq-002, sampled from a severe hospital outbreak, only mutation D614G could be identified.

### Application of AmpliCoV to Elucidate Virus Transmissions

After diagnosing a SARS-CoV-2-positive patient in a co-working space, a PCR screening revealed two additional PCR-positive cases (Ct values specimen 1 = 18.8, specimen 2 = 27.0, and specimen 3 = 19.2). AmpliCoV was used to generate the genome sequences in 1:30 h in total with percentage genome length coverages of 98.2, 97.5, and 98.2% (mean depth of coverage of 3614, 3681, and 7378). Genome analysis showed that there was no relationship between the three cases, pointing toward independent infection events. Illumina sequencing confirmed the AmpliCoV results with the method-inherent delay. In direct comparison of the genomes generated by Illumina sequencing and MinION AmpliCoV sequencing, no SNPs could be identified, except for single indels in homopolymer regions, which were corrected within the bioinformatics generation of the consensus sequences.

## Discussion

Whole-genome sequencing of SARS-CoV-2 has been an integral part of outbreak investigations since the identification of the first COVID-19 cases. As of 17 March 2021, more than 792,347 genomes of SARS-CoV-2 have been made available on GISAID, and additional genomes are being added daily. Recently, the WHO has outlined current and future benefits of real-time virus genome data sharing during outbreaks (World Health Organization [WHO], 2017). Genomic surveillance can be relevant for several outbreak investigations, including virus diversity, virulence, transmission routes, possible mutations, identification of hotspots of transmission, improvement of molecular diagnostics tools and therapeutics. The relevance of whole-genome sequencing has been shown during several viral outbreaks (Grubaugh et al., 2019). For example, the sequencing and analysis of more than 1,600 Ebola virus genomes during the epidemic in West Africa in 2013–2016 has shown mutations in the virus genome compatible with increased fitness because of host adaption (Diehl et al., 2016; Urbanowicz et al., 2016). During the Zika outbreak in 2016, sequencing and phylogenetic analyses of Zika virus from patients and mosquitoes could show multiple introduction events (Grubaugh et al., 2017). Also in the current outbreak of SARS-CoV-2, the comprehensive collection of whole-genome sequences that is updated daily has enabled the calculation and analysis of phylogenetic trees, receptor binding surveillance, important updates on *in silico* binding of diagnostic assays, 3D protein structure modeling, and investigation of potential drug targets^6^. Recently, SARS-CoV-2 mutations have been identified in various regions that could pose problems to vaccine efficiency or display increased virulence and transmissibility (European Centre for Disease Prevention and Control [ECDC], 2020; Koopmans, 2021; Oude Munnink et al., 2021).

However, sequencing of viral genomes directly from patient samples is often hindered by the high amount of human background sequences. To achieve full and deep coverage of the viral genomes, the labor-, time-, and cost-intensive generation of many million reads is often necessary. To increase the number of generated viral reads, preceding culturing of the viral material on cells can be performed. For the enrichment of SARS-CoV-2, a hybridization-based approach with nucleotide baits has been invented, but the method has not yet been validated and, regarding time, is more extensive than other approaches, for example amplification-based enrichment methods (Nasir et al., 2020).

In this study, we report the development of a multiplex-PCR and MinION sequencing approach (AmpliCoV) for whole-genome sequencing of SARS-CoV-2. We have shown that AmpliCoV provides sensitive (up to relative concentrations of a Ct value of 35 with the Sequencing by Ligation Kit) whole-genome sequencing of SARS-CoV-2 and sequencing times of only 30 min until almost complete genome coverage (shown for Ct 24.7). For high and medium virus concentrations (Ct < 32), library preparation can be performed with the Rapid Barcoding Kit with only 10 min of hands-on time. Whole-genome sequencing of SARS-CoV-2 directly from patient material could be shown successfully, eliminating time-consuming culturing of the material or deep sequencing of many millions of reads to achieve appropriate coverage of the genome.

However, there are some considerations to be made when using the AmpliCoV whole-genome sequencing approach. First, sequencing of the sample with the Sequencing by Ligation Kit and a relatively low virus concentration (Ct 35) required a sequencing time of 16:08 h until a relatively low coverage (92.2%) of the genome was achieved. Sequencing times decreased significantly for samples with higher virus concentration (09:34 min–05:55 h for average Ct values from 25–30, genome coverage of 95.3–99.2%). However, as all samples were sequenced in a realistic setting of routine diagnostics with different sample qualities, levels of multiplexing, and numbers of active pores, the sequencing times were not continuously in correlation with the virus concentration (e.g., sample AC_05 with a Ct of 17.7 and a sequencing time of 40:09 min (genome coverage 95.9%). Although sequencing with the Rapid approach can be a flexible alternative to ligation sequencing, sequencing times were longer compared to the samples prepared with the Sequencing by Ligation Kit (01:39–02:42 h compared to 31:16–53:15 min). In direct comparison the coverage of genomes prepared with the Rapid kit was slightly, but not significantly higher than for samples prepared with the Sequencing by Ligation Kit. For both methods, at higher Ct values, drop out of specific amplicons was noticed, not related to length of amplicons. However, for reasons of comparison only 50,000 reads were analyzed. In most cases, analyzing 100,000 reads per sample was sufficient for almost complete (>98%) genome percentage coverages. Genome percentage coverage of clinical samples with low viral load could also be increased by normalizing the libraries before sequencing (data not shown). For elucidating an event of virus transmission, the accuracy of genomes obtained by AmpliCoV sequencing was as with those genomes generated by Illumina sequencing, allowing the reliable identification of SNPs. Furthermore, no differences in the reconstructed genomes were observed after sample preparation of the same sample with the Ligation Kit and the Rapid Kit. This is crucial, as the Rapid kit preparation can be prone to mis-assignment and crossover of barcodes, leading to false positives called variants. Obtained coverage was also high enough to draw conclusions about mutations that occurred recently in Denmark, the United Kingdom, and elsewhere. We could show that sequencing of the S gene was possible, although sequencing of a sample with low viral load (Ct 32.2) resulted in low coverage depth of one amplicon, with successful identification of only 8 of 9 mutations. However, AmpliCoV can be a superior method to mutation screening with PCR.

A number of amplicon-based sequencing approaches are currently being applied for SARS-CoV-2 sequencing, foremost the Artic network protocol (Freed et al., 2020; Gohl et al., 2020; Hourdel et al., 2020). Although it has been shown for all approaches that generation of almost complete SARS-CoV-2 genomes is possible with less than 100,000 reads, the sequencing of complete genomes can be challenging for samples within the routine diagnostics, where concentration and quality of the samples can be low. Although not substantially new, here we have developed an alternative amplicon-based approach which was validated on a number of patient samples (*n* = 36) that have been sequenced in the frame work of realistic routine diagnostics of SARS-CoV-2. We were able to generate almost complete SARS-CoV-2 genomes, although samples were sequenced with different levels of multiplexing and also on low quality Flow Cells, given the low number of active pores. In comparison to the Artic protocol, our approach offers flexible usage of primer pool composition (e.g., sub-pooling of primers targeting only the S gene for specific questions), reduced PCR times (30 s of annealing compared to 5 min) and reduced hands-on time (compatible with the Rapid Sequencing Kit). Furthermore, with an increasing number of mutations in the SARS-CoV-2 genome over time, mismatches between primer binding sites and primers might hinder amplification of some amplicons. To reduce this risk, we have designed the primers in conserved regions (if possible) between SARS-CoV and SARS-CoV-2, which might be less susceptible to mutation and are still matching the huge majority of known sequences according to *in silico* testing. The amplification of up to 81.71% of regions of the SARS-CoV genome shows that primer binding sites with two mismatches to the primer sequences provide amplification of the correlated amplicon. With the further progressing mutation of the SARS-CoV-2 genome, additional primers can be included into the primer panel, if necessary. However, in comparison to mutant-specific PCR assays, the AmpliCoV approach will in all probability also identify unexpected mutations that would be missed by specific PCR assays, usually failing when mismatches occur in relevant positions of primers or probes.

## Conclusion

Genome sequencing has been shown to be an irreplaceable tool for real-time outbreak investigation, including initial pathogen detection and characterization, transmission chain tracking, and protein modeling. In particular, the last months have demonstrated that the identification of specific mutants is of highest importance. We have established and evaluated a whole-genome sequencing method for SARS-CoV-2, called AmpliCoV, which eliminates time-consuming culturing of the patient material or bait hybridization. Furthermore, personal data protection can be ensured, as the amount of sequenced host DNA is minimized markedly. In combination with the affordable and field-deployable Oxford Nanopore MinION, whole-genome sequencing can be performed not only by highly equipped laboratories, but also by laboratories with limited budgets and in remote areas, making the contribution to investigating the SARS-CoV-2 outbreak possible.

## Data Availability Statement

The datasets presented in this study can be found in online repositories. The names of the repository/repositories and accession number(s) can be found below: https://www.ncbi.nlm.nih.gov/bioproject/PRJEB42647.

## Ethics Statement

The studies involving human participants were reviewed and approved by Berliner Ärztekammer (#Eth 20/40). The patients/participants provided their written informed consent to participate in this study.

## Author Contributions

AB designed the assay, established AmpliCoV, analyzed the data, and wrote the manuscript. S-LU, SU, SF, and DS analyzed the samples and the data. RO showed the proof of concept for clinical specimens for AmpliCoV. MC provided clinical specimens carrying various mutations. JM analyzed the suspected SARS-CoV-2 specimens. LS and AN conceptualized the AmpliCoV approach and wrote the manuscript. All authors contributed to the article and approved the submitted version.

## Conflict of Interest

MC was employed by company LADR Laboratory Group Dr. Kramer & Colleagues. The remaining authors declare that the research was conducted in the absence of any commercial or financial relationships that could be construed as a potential conflict of interest.

## Footnotes

1 https://covid19.who.int/

2 https://artic.network/ncov-2019

3 https://github.com/rrwick/Porechop

4 https://github.com/nanoporetech/medaka

5 https://bedtools.readthedocs.io/en/latest/

6 https://www.gisaid.org/
