# Supplementary material for: AmpliCoV: Rapid Whole-Genome Sequencing Using Multiplex PCR Amplification and Real-Time Oxford Nanopore MinION Sequencing Enables Rapid Variant Identification of SARS-CoV-2
Source: Front Microbiol. 2021 Jul 1;12:651151. doi: 10.3389/fmicb.2021.651151 (PMC8281033; doi:10.3389/fmicb.2021.651151)
Supplement: Supplementary file 1 [file Table_1.DOCX]

**Supplementary Table 1.** Primer used in panel A and B for AmpliCoV sequencing of SARS-COV-2 genomes. Primers in bold are used in two-fold concentration.

| Panel A | sequence | Panel B | sequence |
| --- | --- | --- | --- |
| A_AmpliCoV_1 | CGACGAGCTTGGCACTGA | B_AmpliCoV_1 | AAACGAACTTTAAAATCTGTGTGGC |
| A_AmpliCoV_2 | TGGACATTCCCCATTGAAGGT | B_AmpliCoV_2 | GGGCCACAGAAGTTGTTATCG |
| A_AmpliCoV_3 | GCAGACGGGCGATTTTGTTA | B_AmpliCoV_3 | CTGCCGTGAACATGAGCATG |
| A_AmpliCoV_4 | TGTGGAAGCAGAAAAAGATGCC | B_AmpliCoV_4 | CGGCAAGACTATGCTCAGGT |
| A_AmpliCoV_5 | ACTGTGAAAGGTTTGGATTATAAAGCA | B_AmpliCoV_5 | AAGTGTGCCTATTGGGTTCC |
| A_AmpliCoV_6 | GCGGCCTTCTGTAAAACACG | **B_AmpliCoV_6** | ACTCCAAAAAGAGAAAGTCAACATCA |
| **A_AmpliCoV_7** | GATGTTCACATCTGATTTGGCTACT | **B_AmpliCoV_7** | TCACCAATATTCCAGGCACCT |
| **A_AmpliCoV_8** | TCCCTTTGAGTGCGTGACAA | B_AmpliCoV_8 | AGAGGCTGCTCGTGTTGTAC |
| A_AmpliCoV_9 | GCTCCATTGGTTGGTACACC | B_AmpliCoV_9 | CACCACCTGTAATGTAGGCCA |
| A_AmpliCoV_10 | TGACAGCATCTGCCACAACA | B_AmpliCoV_10 | AGTTAGCCACTGCGAAGTCA |
| A_AmpliCoV_11 | GTGCCACTTCTGCTGCTCTT | B_AmpliCoV_11 | TGGCTTTGTGTGCTGACTCT |
| A_AmpliCoV_12 | CCAGCTGATAATAATGGTGCAAGT | B_AmpliCoV_12 | CCGCCTTTGAGTGTGAAGGT |
| A_AmpliCoV_13 | CGCACAAATGTCTACTTAGCTGT | B_AmpliCoV_13 | AGTACTTAATGAGAAGTGCTCTGC |
| A_AmpliCoV_14 | CCCTCTTGAACAACATCACCCA | B_AmpliCoV_14 | CTGATTGTCCTCACTGCCGT |
| **A_AmpliCoV_15** | TGGTGGCACTACTGAAATGC | B_AmpliCoV_15 | TCTTCATGTTGTCGGCCCAA |
| **A_AmpliCoV_16** | AGGAACCAGCAAGTGAGATGG | **B_AmpliCoV_16** | TGCTTATGAAAATTTTAATCAGCACGA |
| A_AmpliCoV_17 | CGCAAGTTGTGGACATGTCA | **B_AmpliCoV_17** | TTGTTTTCTCTGTTCAACTGAAGGT |
| A_AmpliCoV_18 | TGGCATGTTGAAACAAGTAACTCA | B_AmpliCoV_18 | TGGAAGAAACTAAGTTCCTCACAGA |
| A_AmpliCoV_19 | TGGTACATTTACTTGTGCTAGTGAGT | B_AmpliCoV_19 | CTGACCCGGGTAAGTGGTTAT |
| A_AmpliCoV_20 | TCAATAGCCACCACATCACCAT | B_AmpliCoV_20 | AGCACTGTCTTTGCCTCCTC |
| A_AmpliCoV_21 | GTCAGAGGACGCGCAGG | B_AmpliCoV_21 | TCTCAAAGTGCCAGCTACAGT |
| **A_AmpliCoV_22** | ACTACCGAAGTTGTAGGAGACATT | B_AmpliCoV_22 | AACATCAGCTCCATCCAAATAAGTTG |
| **A_AmpliCoV_23** | CAACTTTGTTAAGAAAAGGCTTAGCA | B_AmpliCoV_23 | ATCCACCTGCTCTACAAGATGC |
| **A_AmpliCoV_24** | ACCGTGTTTGTACTAATTATATGCCTT | B_AmpliCoV_24 | GCACCGTCTATGCAATACAAAGT |
| **A_AmpliCoV_25** | AACACCTAAAGCAGCGGTTG | B_AmpliCoV_25 | AAGAAACCTGCTTCAAGAGAGCT |
| A_AmpliCoV_26 | ACCACTAAGACAAACACTACAAGGT | B_AmpliCoV_26 | TCTGTGTGGCCAACCTCTTC |
| A_AmpliCoV_27 | GTTGCAGAGTGGTTTTTGGCA | B_AmpliCoV_27 | TGCTACTCATGGTTTAGCTGCT |
| A_AmpliCoV_28 | ATGGATGGAACCATTCTTCACTGT | B_AmpliCoV_28 | AGTATTCTTTGCTATAGTAGTCGGCA |
| A_AmpliCoV_29 | GGTGATAGTGCGGAAGTTGC | B_AmpliCoV_29 | GTGTCGGTAAATTTTGTCTAGAGGC |
| A_AmpliCoV_30 | ACAATTTTACCACCCTTAAGTGCT | B_AmpliCoV_30 | AGCTGAAATCGGGGCCATTT |
| A_AmpliCoV_31 | CTTGCCCATTGATTGCTGCA | B_AmpliCoV_31 | AAGTTGCGAGAGACTTGTCACT |
| A_AmpliCoV_32 | AAGTGCCGTGCCTACAGTAC | B_AmpliCoV_32 | AGTTCAGCTTCTGCAGTTGC |
| A_AmpliCoV_33 | ATCGTAGTAACATGCCTTGCCT | B_AmpliCoV_33 | AGCAAAAAGTCACAACATTGCTTTG |
| A_AmpliCoV_34 | GCTAGTTGTATCCATTGCTCCAC | B_AmpliCoV_34 | GGCACGACAAAACCCACTTC |
| A_AmpliCoV_35 | CCCATCTGGTAAAGTTGAGGGT | B_AmpliCoV_35 | CCTTGAAGGTTCTGTTAGAGTGGT |
| A_AmpliCoV_36 | TGCACCTCTGAAGACATGCT | B_AmpliCoV_36 | ACTGGTGTTAAACAGAGTACAGTGA |
| A_AmpliCoV_37 | ACCAACACTACCACATGAACCA | B_AmpliCoV_37 | GCGCTGTGCACCTTTTTGT |
| A_AmpliCoV_38 | ACTGTAATAGTTGTGTCCGTACCA | B_AmpliCoV_38 | TGCGTAGTGATGTGCTATTACCT |
| A_AmpliCoV_39 | TCTCAATCGATTTACCACAACTCT | B_AmpliCoV_39 | TGAACATTACCAGCCTGTACCA |
| A_AmpliCoV_40 | ACCTCTAACACAAGACCATGTTGA | B_AmpliCoV_40 | TTAGGTGTCTTAGGATTGGCTGT |
| A_AmpliCoV_41 | ACAAAGACCATTGAGTACTCTGGA | B_AmpliCoV_41 | ACCAATGTGCTATGAGGCCC |
| A_AmpliCoV_42 | TCATTGCAAAAGCAGACATAGCA | B_AmpliCoV_42 | AACGGCAATTCCAGTTTGAGC |
| A_AmpliCoV_43 | TGGTGCTAGGAGAGTGTGGA | B_AmpliCoV_43 | ACACCACTGGTTGTTACTCACA |
| A_AmpliCoV_44 | TGGACACATTGAGCCCACAA | B_AmpliCoV_44 | CCTCTGGCCAAAAACATGACA |
| A_AmpliCoV_45 | CATGCAGGGTGCTGTAGACA | B_AmpliCoV_45 | CTACTCCCACCCAAGAATAGCA |
| A_AmpliCoV_46 | GGGAACACAACCATCTCTTGC | B_AmpliCoV_46 | CACCATTAGCAACAGCCTGC |
| A_AmpliCoV_47 | AGGGCCAATTCTGCTGTCAA | B_AmpliCoV_47 | AATTTGACCGTGATGCAGCC |
| A_AmpliCoV_48 | GTGATTGGTTGTCCCCCACT | B_AmpliCoV_48 | TGTAGTACCGGCAGCACAAG |
| A_AmpliCoV_49 | CAACTTGTGCTAATGACCCTGTG | B_AmpliCoV_49 | AATGCAACAGAAGTGCCTGC |
| A_AmpliCoV_50 | AAAGCATAGACGAGGTCTGCC | B_AmpliCoV_50 | ACTTACACCGCAAACCCGTT |
| A_AmpliCoV_51 | TGGTAACTGGTATGATTTCGGTGA | B_AmpliCoV_51 | ACATGGTACCACATATATCACGTCA |
| A_AmpliCoV_52 | CAGGTAGTGGAGTTCCTGTTGT | B_AmpliCoV_52 | TGCAGTTAAAGCCCTGGTCAA |
| A_AmpliCoV_53 | TTGGGTGGTATGTCTGATCCC | B_AmpliCoV_53 | TTGACACTGACTTAACAAAGCCTT |
| A_AmpliCoV_54 | ACTGTTTGGATGACAGATGCATTC | B_AmpliCoV_54 | ACAACACCTAGCTCTCTGAAGTG |
| A_AmpliCoV_55 | GTGCGTTTATCTAGTAATAGATTACCAGA | B_AmpliCoV_55 | TGTGTATGCTGCTGACCCTG |
| A_AmpliCoV_56 | ACCAAGTCATCGTCAACAACCT | B_AmpliCoV_56 | TGCGAAAAGTGCATCTTGATCC |
| A_AmpliCoV_57 | AGCAAGAACAAGTGAGGCCA | B_AmpliCoV_57 | CAATAGCCGCCACTAGAGGAG |
| A_AmpliCoV_58 | GAAATGGTCATGTGTGGCGG | B_AmpliCoV_58 | AAGCAGTTGTGGCATCTCCT |
| A_AmpliCoV_59 | TGAGAGCAAAATTCATGAGGTCCT | B_AmpliCoV_59 | ACAACACAGACTTTATGAGTGTCTCT |
| A_AmpliCoV_60 | GGGCCGGCTGTTTTGTAGA | B_AmpliCoV_60 | TCTGACGATGCTGTTGTGTGT |
| A_AmpliCoV_61 | AGCTCATACCTCCTAAGTAAAGTTGA | B_AmpliCoV_61 | AGCTAAAGACACGAACCGTTCA |
| A_AmpliCoV_62 | TGTGCTAATGGACAAGTTTTTGGTT | B_AmpliCoV_62 | TGAAAGACATCAGCATACTCCTGA |
| A_AmpliCoV_63 | TGTGACTGGACAAATGCTGGT | B_AmpliCoV_63 | CGACCATGTCATATCAACATCACA |
| A_AmpliCoV_64 | TGCTCTTGTGGCACTAGTGT | B_AmpliCoV_64 | TCTGTCAGACAGCACTTCACG |
| A_AmpliCoV_65 | ACTCATCTGAGATATTGAGTGTTGGG | B_AmpliCoV_65 | TGCTGTTGTTTACCGAGGTACA |
| **A_AmpliCoV_66** | TGGTACTGGTAAGAGTCATTTTGC | B_AmpliCoV_66 | CATAGTGCATCAACAGCGGC |
| **A_AmpliCoV_67** | TGAAATATTCTGGTTCTAGTGTGCC | B_AmpliCoV_67 | ATTGGCGACCCTGCTCAATT |
| A_AmpliCoV_68 | ACAGAACTTCCTTCCTTAAAGAAACC | B_AmpliCoV_68 | AGCCCTGTGATGAATCAACAGT |
| **A_AmpliCoV_69** | ACGCATGATGTTTCATCTGCA | B_AmpliCoV_69 | GCTATTACCAGAGCAAAAGTAGGC |
| **A_AmpliCoV_70** | TGTAAAGTTGCCACATTCCTACG | B_AmpliCoV_70 | CAGGCGGTGGTTTAGCACTA |
| A_AmpliCoV_71 | TGGCATACCTAAGGACATGACC | B_AmpliCoV_71 | ACTTCCTTGGAATGTAGTGCGTA |
| A_AmpliCoV_72 | AACTCAAAGCCATGTGCCCA | B_AmpliCoV_72 | GTGACACACTTAAAAATCTCTCTGACA |
| A_AmpliCoV_73 | TGCGGCTTGTAGAAAGGTTCA | B_AmpliCoV_73 | CCAATGTCGTGAAGAACTGGG |
| A_AmpliCoV_74 | GCTGGTGTGTGGAATGCATG | B_AmpliCoV_74 | TGTCACTACAAGGCTGTGCA |
| A_AmpliCoV_75 | CATGTGAGTCTCATGGAAAACAA | **B_AmpliCoV_75** | TGCCACACATTCTGACAAATTCA |
| A_AmpliCoV_76 | GCCCAAAGCTCAAATGCTACA | **B_AmpliCoV_76** | ACAGCACCACCTAAATTGCA |
| **A_AmpliCoV_77** | GAAATGCCCGTAATGGTGTTCTT | B_AmpliCoV_77 | ACACAAAAGTTGATGGTGTTGATGT |
| **A_AmpliCoV_78** | TGAACCTGTTTGCGCATCTG | B_AmpliCoV_78 | GTTGGACAACACCATCAACTTTCTT |
| A_AmpliCoV_79 | TCTGTAGTTTCTAAGGTTGTCAAAGTG | B_AmpliCoV_79 | GGTGGTTTACATCTACTGATTGGAC |
| A_AmpliCoV_80 | AGAACCAGCACCAAAATGTATAACTC | B_AmpliCoV_80 | TAGGCATAGCAACACCCGG |
| A_AmpliCoV_81 | GAGTTGCACCAGGTACAGCT | B_AmpliCoV_81 | TGCTATTAGAAAAGTGTGACCTTCAAA |
| A_AmpliCoV_82 | AAAGACATAACAGCAGTACCCCTT | B_AmpliCoV_82 | TGTACAGTTGCACAATCACCAATC |
| A_AmpliCoV_83 | CATGTCTCTGGGACCAATGGT | B_AmpliCoV_83 | GGCAAACCACGCGAACAAAT |
| A_AmpliCoV_84 | TGGTGTTTATTTTGCTTCCACTGA | B_AmpliCoV_84 | AGTAGGGACTGGGTCTTCGAA |
| **A_AmpliCoV_85** | CCTGAGGGAGATCACGCAC | B_AmpliCoV_85 | TCAGCCTTTTCTTATGGACCTTGA |
| **A_AmpliCoV_86** | TAATAAGCTGCAGCACCAGC | B_AmpliCoV_86 | CAGATGCAAATCTGGTGGCG |
| A_AmpliCoV_87 | ACCCTCTCTCAGAAACAAAGTGT | B_AmpliCoV_87 | CAAATCGCTCCAGGGCAAAC |
| A_AmpliCoV_88 | TTCCAAGCTATAACGCAGCCT | B_AmpliCoV_88 | CTTTTTAGGTCCACAAACAGTTGCT |
| A_AmpliCoV_89 | CTATCAGGCCGGTAGCACAC | B_AmpliCoV_89 | TCAACTTACTCCTACTTGGCGT |
| A_AmpliCoV_90 | CAGCCTGCACGTGTTTGAAA | B_AmpliCoV_90 | CAGCTATTCCAGTTAAAGCACGG |
| A_AmpliCoV_91 | GGTGATTCAACTGAATGCAGCA | B_AmpliCoV_91 | TGCACAAAAGTTTAACGGCCT |
| A_AmpliCoV_92 | ACAGTGCAGAAGTGTATTGAGCA | B_AmpliCoV_92 | GCACTTCAGCCTCAACTTTGT |
| A_AmpliCoV_93 | ACACGCTTGTTAAACAACTTAGCT | B_AmpliCoV_93 | CAACTGCTCCTGCCATTTGTC |
| A_AmpliCoV_94 | TGTTACAAACCAGTGTGTGCC | B_AmpliCoV_94 | ATATTTATCTAACTCCTCCTTGAATGAGTC |
| A_AmpliCoV_95 | TGTGTCTGGTAACTGTGATGTTGT | B_AmpliCoV_95 | TGATTTAGGTGACATCTCTGGCA |
| A_AmpliCoV_96 | AGCCAGCTATAAAACCTAGCCA | B_AmpliCoV_96 | TGCAGCAGGATCCACAAGAA |
| A_AmpliCoV_97 | TGCTGTATGACCAGTTGCTGT | B_AmpliCoV_97 | CTTGCTGTTTTTCAGAGCGCT |
| A_AmpliCoV_98 | ACGAGCAAAAGGTGTGAGTAAAC | B_AmpliCoV_98 | ACAGTCTTTTACTCCAGATTCCCA |
| A_AmpliCoV_99 | GGTGATGGCACAACAAGTCC | B_AmpliCoV_99 | TGAGTACAGACACTGGTGTTGAA |
| A_AmpliCoV_100 | AGTCGTCGTCGGTTCATCAT | B_AmpliCoV_100 | AGCAGTACGCACACAATCGA |
| A_AmpliCoV_101 | TTCGTTTCGGAAGAGACAGGT | B_AmpliCoV_101 | AACCTTCTTTTTACGTTTACTCTCGT |
| A_AmpliCoV_102 | TCGTTTAGACCAGAAGATCAGGA | B_AmpliCoV_102 | CCATAACAGCCAGAGGAAAATTAACT |
| A_AmpliCoV_103 | CCATGGCAGATTCCAACGGT | B_AmpliCoV_103 | GGATCACCGGTGGAATTGCT |
| A_AmpliCoV_104 | CAGCGTCCTAGATGGTGTCC | B_AmpliCoV_104 | CCTGAGTCACCTGCTACACG |
| A_AmpliCoV_105 | GCTACATCACGAACGCTTTCT | B_AmpliCoV_105 | ACATACGAGGGCAATTCACCA |
| A_AmpliCoV_106 | GCCGTCAGGACAAGCAAAAG | B_AmpliCoV_106 | GGACACGGGTCATCAACTACA |
| A_AmpliCoV_107 | TGTCACGCCTAAACGAACATG | B_AmpliCoV_107 | GAATTGTGCGTGGATGAGGC |
| A_AmpliCoV_108 | TTCGCTGATTTTGGGGTCCA | B_AmpliCoV_108 | GCTTCTGGCCCAGTTCCTAG |
| A_AmpliCoV_109 | ACCCAATAATACTGCGTCTTGGT | B_AmpliCoV_109 | GCCAACAACAACAAGGCCAA |
| A_AmpliCoV_110 | TGGGTTTGTTCTGGACCACG | B_AmpliCoV_110 | ACGTACTGCCACTAAAGCATACA |
| A_AmpliCoV_111 | CATTCCCACCAACAGAGCCT | B_AmpliCoV_111 | AGGCTTGAGTTTCATCAGCCT |
| A_AmpliCoV_112 | AAATTAATTTTACACATTAGGGCTCTTCC |  |  |
| A_AmpliCoV_113 | AGCTATTAAAATCACATGGGGATAGC |  |  |
